# Characterization and *In Silico* Analysis of Pregnancy-Associated Glycoprotein-1 Gene of Buffalo *(Bubalus bubalis)*

**DOI:** 10.4061/2011/436138

**Published:** 2011-02-06

**Authors:** Jerome A., S. K. Singh, S. K. Agarwal, Mohini Saini, Ashwin Raut

**Affiliations:** ^1^Division of Animal Reproduction, Indian Veterinary Research Institute, Izatnagar, Bareilly 243 122, India; ^2^Centre for Wildlife, Indian Veterinary Research Institute, Izatnagar, Bareilly 243 122, India; ^3^Division of Animal Biotechnology, Indian Veterinary Research Institute, Izatnagar, Bareilly 243 122, India

## Abstract

Pregnancy-Associated Glycoproteins (PAGs) are trophoblastic proteins belonging to the Aspartic proteinase family secreted by different placental cells of many mammalian species. They play a pivotal role in placentogenesis, foetomaternal unit remodeling, and implantation. The identification of the genes encoding those proteins will be helpful to unravel the intricate embryogenomic functions during pregnancy establishment. Considering importance of these proteins, the present study was undertaken to characterize the pregnancy associated glycoprotein-1 gene of buffalo. An 1181 base pairs buffalo Pregnancy-Associated Glycoprotein PAG-1 gene was PCR amplified from the RNA obtained from the fetal cotyledons. BLAST analysis of the buffalo PAG-1 sequence retrieved a total of 20 cattle, 5 goat, and 4 sheep PAG sequences, exhibiting more than 80% similarity. Buffalo PAG-1 gene contained an uninterrupted open reading frame of 1140 base pairs encoding 380 amino acids that possess a 15 amino acid signal peptide and mature peptide of 365 amino acids. The phylogenetic study of the buffalo PAG-1 gene revealed buffalo PAG-1 is more related to cattle, goat, and sheep PAG-1 sequences. By this study characterization of buffalo PAG-1 gene and its evolutionary relationship was deduced for the first time.

## 1. Introduction

Pregnancy is established and maintained by the two-way communication between the conceptus and the mother. These intricate dialogues which are initiated after fertilization are crucial as these signals are considered potential markers for effective placental remodeling, pregnancy recognition, and successful implantation. These interactions between the conceptus and maternal system emphasize the importance of both the components in maternal recognition of pregnancy and embryonic development [1]. These important signals to the maternal system to sustain pregnancy are mediated by numerous molecules which include steroid hormones, peptide hormones, cytokines, and growth factors [1, 2]. 

Conceptus-derived substances are considered to be precise and reliable markers of pregnancy and fetal well-being. Pregnancy-associated glycoproteins are one such large family of protein molecules produced by conceptus for the recognition by the mother. Pregnancy-associated glycoproteins (PAGs) are acidic glycoprotein belonging to the Aspartic Proteinase superfamily sharing more than 50% amino acid sequence identity with Pepsin, Cathepsin D, and E [3, 4]. 

Pregnancy-associated glycoproteins (PAGs) form very large family of glycoproteins; nearly 22 different PAGs in ruminants have been identified at different stages of gestation [5]. Pregnancy-Associated Glycoprotein-1 (PAG-1) also known as Pregnancy Specific Protein B (PSPB), PSP-60, and SBU3, is secreted by the binucleate cells of the conceptus trophectoderm [6]. PAG-1 is detectable in maternal blood soon after implantation as binucleate cells migrate from the trophectoderm and fuse with uterine epithelial cells and hence it is considered as a potential signal from the conceptus [7]. The products of binucleate cells in maternal circulation have also been reported to be associated with placental mass, fetal number, twins, and neonatal birth weight in cattle [8, 9]. 

The Pregnancy-associated glycoproteins (PAGs) are multigene family expressed in placenta of eutherian mammals and their expression varies spatially as well as temporally during gestation [10]. Multiple PAG genes have been cloned and identified in many domestic animals such as cattle, sheep [5], goat [11], pig [12], and wild ruminants' species [5]. Based on the evolutionary study and phylogenetic linkage bovine pregnancy associated glycoproteins family has been segregated as ancient (bovine PAG-2, bovine PAG-8) and modern (bovine PAG-1) [13–15]. But there is no report on the characterization and phylogenetic analysis of pregnancy-associated glycoprotein-1 gene of buffalo. Moreover, identification of gene encoding buffalo Pregnancy-Associated Glycoprotein-1 may provide an avenue for producing recombinant protein which will be helpful to develop diagnostics for early pregnancy diagnosis and marker for embryonic development [16]. Considering the importance of the gene in embryogenesis, the present study was designed to characterize and analyze pregnancy-associated glycoprotein-1 (PAG-1) gene phylogenetic lineage.

## 2. Materials and Methods

### 2.1. Sample Collection and RNA Isolation

Buffalo placentae were collected from local abattoir. The stage of pregnancy was estimated by measurement of crown-rump length. Placental cotyledons were collected from day 60 of pregnancy. Total RNA was isolated from fetal cotyledons using TRI reagent (Ambion, USA) following manufacture's instructions. The integrity of the extracted RNA was checked by agarose gel (1%) electrophoresis and visualization of the gel under UV light after staining with ethidium bromide. The purity of the obtained RNA was checked by means of spectrophotometric readings at OD_260_/OD_280_.

### 2.2. cDNA Synthesis and Buffalo PAG-1 Gene Amplification

RNA from the fetal cotyledons was reverse-transcribed into cDNA with reverse transcriptase (Qiagen, Germany), oligo (dT) primers, and 500 *μ*M dNTPs at 37°C for 1 hour. On the basis of available PAG-1 sequences from cattle (GenBank Acc. No. M73962; NM 174411), goat (GenBank Acc. No. AF191326), sheep (GenBank Acc.No. M73961), pig (GenBank Acc. No. L34360), white-tailed deer (GenBank Acc. No. AY509865), and zebra (GenBank Acc.No. AF036952) buffalo gene-specific primers were designed using Primer select programme of DNA star software. The primers were forward (PAG-1/start F, 5′-GGATCCAGGAAATAAACATGAAGTG-3′ and PAG-1/stopR, 5′-TTACTGAAC CACTCYMAGCATTT-3′). 

PCR amplification was carried out in a total volume of 25 *μ*L of reaction mixture containing approximately 100 ng of cDNA, 10X PCR buffer (100 Mm Tris-Hcl, pH 8.8 at 25°C, 5 pM of forward and reverse primer of each, 2.0 mM MgCl_2_, 200 *μ*M dNTPs, 1.0 U *Taq* DNA Polymerase. The PCR protocol involved an initial denaturation at 94°C for 2 minutes; 30 cycles of denaturation (94°C for 15 seconds), annealing (optimum temperature of 51.3°C for 15 seconds), and extension (74°C for 45 seconds); one cycle of final extension (74°C for 10 minutes). The PCR product was checked by 1% agarose gel electrophoresis.

### 2.3. Buffalo PAG-1 Gene Cloning

PCR amplicons were cloned in cloning vector (PTZ57R/T, InsTAclone, MBI, Fermentas) following the manufacturer's protocol. The 12 *μ*L of ligated product was directly added to 200 *μ*L competent cells, and cells were then immediately transferred on chilled ice for 5 minutes, and SOC was added. The bacterial culture was pelleted and plated on LB agarplate containing Ampicillin (100 mg/mL) added to agarplate at 1 : 1000, IPTG (200 mg/mL) and X-Gal (20 mg/mL) for blue-white screening. Isolation of plasmid from was done using kit's protocol (Biochem Life sciences). Recombinant plasmids were characterized by PCR using gene-specific primers and restriction enzyme digestion.

Restriction enzyme analysis of the plasmid was carried out with *Xba* I (MBI, Fermentas USA) and *Sma* I (MBI, Fermentas USA) enzymes. Double digestion with *EcoR* I (MBI, Fermentas USA) and *Sma* I (MBI, Fermentas USA) restriction enzymes was also performed.

### 2.4. Sequencing Buffalo PAG-1 Gene

 The plasmid containing buffalo PAG-1 cDNA was sequenced using M13 Forward and Reverse primer pair by primer walking in an automated DNA sequence (Sequence Analyzer Version 2.0, ABI Prism, Chromous Biotech, Bangalore). The sequence obtained was subjected to BLAST (http://blast.ncbi.nlm.nih.gov/Blast.cgi) to retrieve PAG-1 sequences of other species. The nucleotide and deduced amino acid sequences of buffalo PAG-1 gene were aligned and compared with other species sequences available in GenBank using Clustal option in MegAlign (Lasergene Software, DNASTAR). The buffalo PAG-1 protein structure was predicted by online SWISS MODEL software. The domain structure, glycosylation sites, and hairpin loop structure were determined by online software like PROSITE (http://www.expasy.ch/pro), SMART (http://smart.embl-heidelberg.de/). The predicted buffalo PAG-1 protein sequence was statistical analysed using SAPS software. Phylogenetic tree based on the evolutionary distances was constructed using MegAlign (Lasergene Software, DNASTAR), based on the nucleic acid and amino acid alignment. Using MEGA 4.1 software the number of synonymous substitution per synonymous site (*dS*) and number of non-synonymous substitution per nonsynonymous sites (*dN*) were estimated, and neutral (*dS* = *dN*), positive (*dN > dS*), or purifying (*dN < dS*) selections were tested with a codon-based Z test using Nei Gojobori method.

## 3. Results and Discussion

### 3.1. Buffalo PAG-1 Transcript

**Figure 1:**
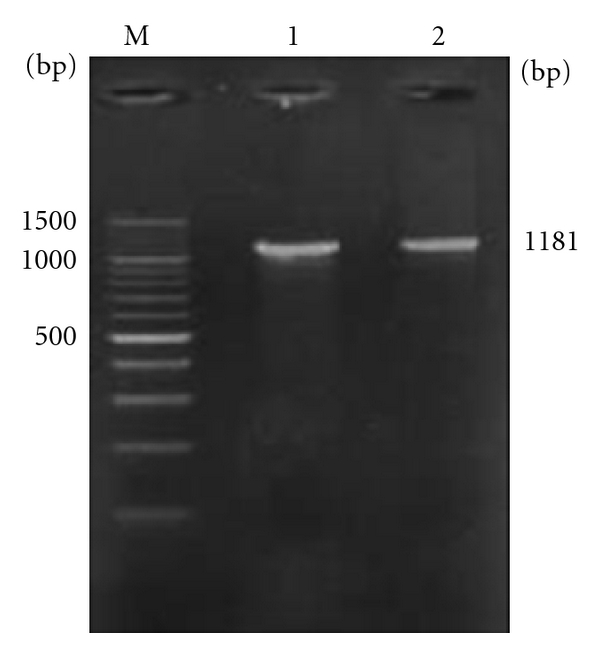
Agarose gel electrophoresis depicting PCR amplified Buffalo PAG-1 gene. Lanes 1 and 2: 1181 bp buffalo PAG-1 gene. Lane M: 100 bp DNA ladder as molecular size marker.

 The concentration of RNA was checked by analyzing OD260/OD280 ratio which was found in the range of 1.8-1.9 indicated the purity of the RNA, and the yield was obtained in range of 3.5–3.8 *μ*g/mL. From the obtained total RNA, cDNA was synthesized and PCR amplification was carried out in 1% agarose gel. Agarose gel electrophoresis revealed 1181 bp PCR product of buffalo PAG-1 gene (Figure 1). Following this restriction enzyme analysis of the plasmid with *Xba* I and *Sma* I enzymes released an insert of 1181 bp. Digestion with *EcoR* I and *Sma* I released as well as digested the insert into fragments of 234 and 947 bp, respectively, confirming the presence of a conserved site for *EcoR*1 for every binucleate specific PAGs.

### 3.2. Sequence Analysis

BLAST analysis of the amplicon sequence retrieved gene sequences of various aspartic proteinase family members which showed greater than 80% similarity. Amongst these, there were 20, 5, and 4 sequences of bovine, caprine, and ovine PAG, respectively, that exhibited more than 80% similarity with buffalo PAG-1 sequence and 5 sequences of white tailed deer which showed less than 80% homology. Accordingly, the sequence was submitted to Genbank as buffalo Pregnancy-Associated Glycoprotein-1 gene under accession number EU815059. The deduced buffalo PAG-1 gene consists of an open reading frame of 1140 nucleotide corresponding to an inferred polypeptide length of 380 amino. Moreover, on translation buffalo PAG-1 gene sequence encodes a signal sequence constituting the first 15 amino acids and a mature peptide of 365 residues. 

Buffalo PAG-1 gene was compared with the members of the aspartic proteinase family, Pregnancy-Associated Glycoprotein gene sequences of cattle and other species.

With the members of aspartic proteinase family buffalo PAG-1 sequence showed a similarity of 62.4, 34.6, 33.8, 32.6, 29.5, and 28.7% with pepsinogen, cathepsin E, chymosin, pepsin, cathepsin D, and rennin, respectively, at the amino acid level. With cattle PAG gene sequences buffalo PAG-1 sequence showed a similarity more than 80% with PAG-1, 3, 16, and less than 80% with PAG-2, 4, 5, 6, 7, 9, 14, 15, 17, 18, 19, 20, and 21. Buffalo PAG-1 sequence showed highest similarity of 89.3% with cattle PAG-1 and lowest of 54.0% with cattle PAG-8. 

### 3.3. Phylogenetic Analysis

**Figure 2:**
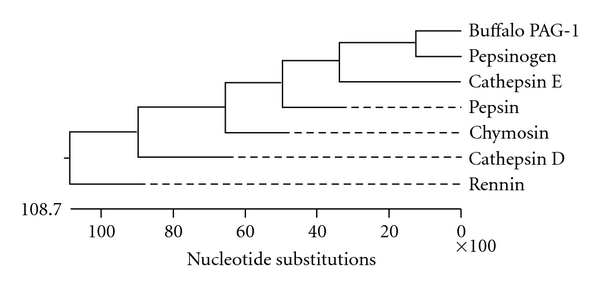
Phylogram depicting the evolutionary relationship of buffalo PAG-1 with members of aspartic proteinase family-based amino acid sequence.

The phylogenetic analysis of the obtained sequence of buffalo PAG-1 gene (GenBank Acc No. EU815059) with members with aspartic proteinase family members revealed that buffalo PAG-1 sequence is more related to pepsinogen and less related with rennin (Figure 2). The degree of similarity was determined comparing the obtained buffalo nucleotide and derived amino acid sequence with available PAG-1 sequences of cattle (GenBank Acc. No. M73962;.NM 17441), goat (GenBank Acc. No. AF191326), sheep (GenBank Acc. No M73961), horse (GenBank Acc. No. L38511), zebra (GenBank Acc. No AF036952), white tailed deer (GenBank Acc. No AY509865), pig (GenBank Acc. No. L34360), and cat (GenBank Acc. No. AF036953). Buffalo PAG-1 showed the percent similarity ranging from 47 to 89% and 39 to 81%, at nucleotide and derived amino acids sequences levels, respectively, with other species PAG-1 sequences. Buffalo PAG-1 sequence showed highest similarity of 89.2% with cattle followed by 75.6% with goat, 74.5% sheep, 72.5% white tailed deer and 52.1% pig, 51.9% cat, 47.3% zebra, and 47.1% horse. 

**Figure 3:**
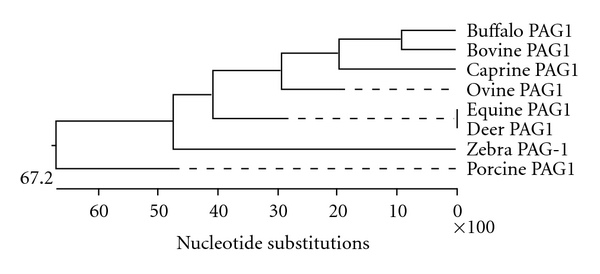
Phylogram depicting the evolutionary relationship of buffalo PAG-1 with other species PAG-1 sequences.

**Figure 4:**
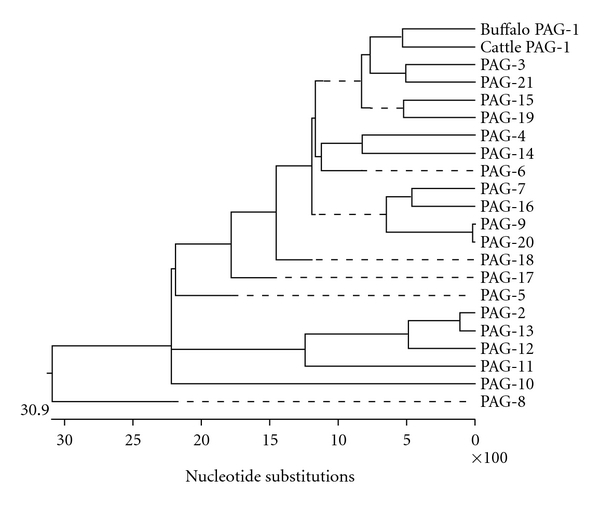
Phylogram depicting buffalo PAG-1 gene relationship with cattle PAG sequences.

Phylograms constructed on the basis of nucleotide and derived amino acids sequences of buffalo PAG-1 sequence with other species showed buffalo, cattle, sheep, goat, horse, white tailed deer, zebra, feline comprise one clade, and pig comprise another. Porcine PAG-1 stands alone and represents an entirely different clade. The PAG-1 sequence of cattle and buffalo belonged to same group and showed more closeness to goat. The PAG-1 of horse, deer, and zebra seems to be an evolutionary connecting link between ruminants and other nonruminants. Horse, deer, and zebra show more evolutionary closeness to each other but far distant from ruminants (Figure 3). Analysis of phylogenetic tree constructed with buffalo PAG-1 and cattle PAG sequences revealed that buffalo PAG-1 gene is more closely related to cattle PAG-1, 3, 21, and 19 both at nucleotides and amino acids level. Cattle PAG-2 group occupies an intermediate position between the ancient aspartic proteinase family members and PAG-1 group. Buffalo PAG-1 as well as other species PAG-1 sequences comprise one clade and show more divergence from the ancient aspartic proteinase family members (Figure 4). Codon-based Z test using the Nei Gojobori method revealed that at 5% level of significance, *dN* is substantially greater than *dS*. Thus, buffalo PAG-1 might have evolved by recently by positive selection (*dN > dS*) among these species. Buffalo and cattle PAG-1 gene showed identical lineage. Goat, sheep, horse, and deer are similar but have different lineage. Pig sequence show dissimilarities suggesting different ancestry.

### 3.4. Predicted Protein Analysis

The predicted buffalo PAG-1 protein sequence was statistically analyzed using SAPS software revealed the total number of negatively and positively charged residues charged residues were 30 and 40, respectively. The atomic composition of the protein consists of Carbon (C) 1970; Hydrogen (H) 3011; Nitrogen (N) 521; Oxygen (O) 539; Sulfur (S) 15 with 6056 atoms. The extinction coefficient is 1.828 M^−1^ cm^−1^, at 280 nm measured in water. The aliphatic index and theoretical pI is 93.32 and 9.10, respectively, with hydropathicity index of 0.071. The estimated half life of buffalo PAG-1 protein is 30 hours with an instability index of 32.12 predicting the protein as stable. 

**Figure 5:**
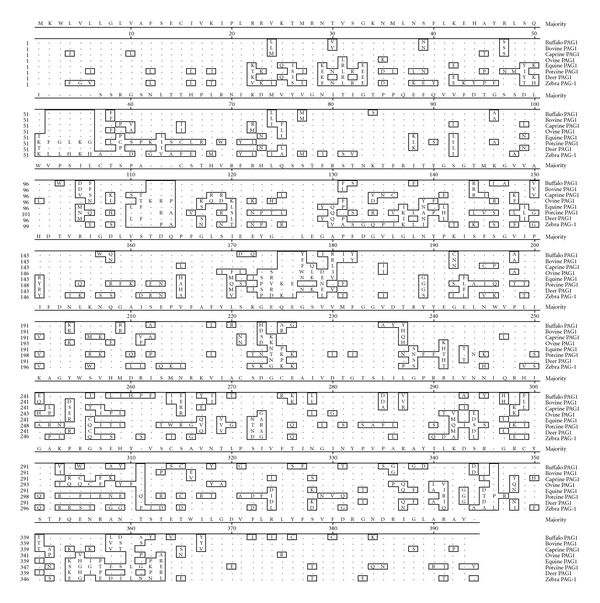
MegAlign report of buffalo PAG-1 amino acid sequence (380 amino acids) with PAG-1 amino acid sequences of other species.

**Figure 6:**
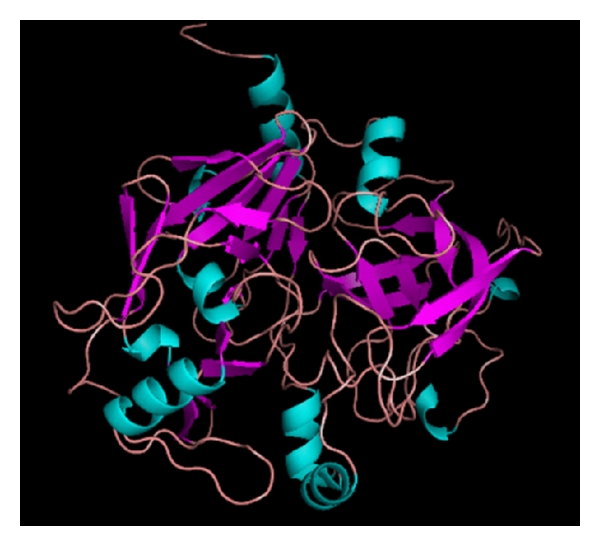
Predicted 3D structure of buffalo PAG-1 protein and its residues.

Sequence of deduced PAG-1 gene of buffalo revealed an insert of 1181 bp. Comparing the buffalo nucleotides and derived amino acids with available PAG-1 sequences of other species confirm buffalo PAG-1 contains an open reading frame of 1140 nucleotides which on translation corresponds to a polypeptide length of 380 amino acids similar to cattle. On translation, buffalo PAG-1 cDNA yields a polypeptide of 380 amino acids with a signal peptide encoded by the first 15 amino acids followed by a mature peptide of rest 365 residues. The signal sequence of the derived buffalo PAG-1 amino acid sequence (MKWLVLLGLVAFSEC) starts and terminates with methionine and cysteine, respectively. This signal sequence is well conserved in buffalo as in bovine and other species [3] (Figure 5). The predicted buffalo PAG-1 protein structure along with its amino acid residues was also deduced (Figure 6). 

 The derived mature peptide sequence consists of well-conserved motif (ISF↓RGS) between the propeptide and the mature molecule. The arrow indicates the site of cleavage between the propeptide and the mature protein. The N-terminal ends of ruminant PAG native proteins predominantly contains the sequence of 3 amino acids, ↓R-D/G-S which is also deduced in buffalo PAG-1-derived amino acid sequence [17, 18]. On comparison of the amino acid sequences of buffalo PAG-1 with other species PAG sequences, it is evident that both amino terminus 91 to 98 (VVFDTGSS) and carboxyl terminus 278–284 (LVDTGTS) are conserved across the domestic species (90–100%) suggesting importance of this region for the diagnostic function of PAG-1 and suggesting that the buffalo PAG-1 has also evolved from same family [3, 19]. Moreover, residues flanking the aspartic acid residues which are considered to be essential for catalytic activity of pepsin are well conserved in buffalo as in other domestic species [20]. Although the PAGs clearly belong to the aspartic proteinases, they are not active proteolytically owing to the key mutations close to the active site due to amino and carboxyl terminal flanking the aspartic acid residues that would likely interfere with the catalytic mechanism. On analysis it was evident that buffalo PAG-1 is not proteolytically active since it possesses key mutation of alanine substitution in place of glycine at the active site as same as boPAG-1 which will displaces a water molecule that normally resides between the two catalytic aspartic acids and is directly involved in the catalytic mechanism [3, 20]. Although PAG-1 group molecules are not proteolytically active, they have retained the characteristic bilobed structure of aspartic proteinase [20]. The catalytic mechanism, which leads to peptide bond cleavage in the middle of the bound peptide, is initiated through nucleophilic attack by a hydroxyl ion supplied by a water molecule strategically positioned between the two aspartic acids [19]. Comparison of buffalo PAG sequence and other species sequence reveals segments of primary structure that are hypervariable and others that are relatively constant. The region between residues 70 and 100 (13–42 pepsin numbering) showed very few substitutions depicting the presence of other conserved sequence [4]. From the alignment study it was evident that buffalo PAG-1 which showed more similarity to cattle PAG-1 will also possess a three-dimensional fold and may have a general affinity for binding peptides with basic residues [20].

**Figure 7:**
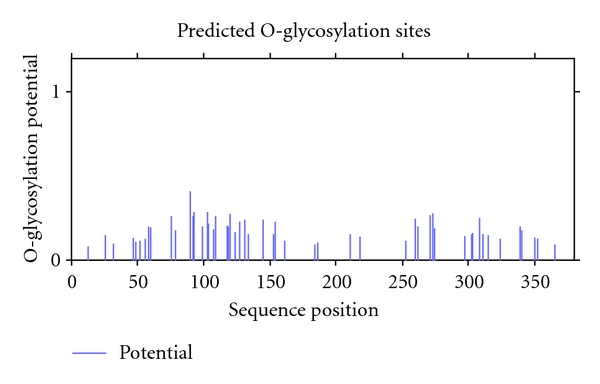
Predicted potential O-glycosylation sites of buffalo PAG-1 protein sequence.

The deduced buffalo PAG-1 amino acid sequence contains microsequences YS (position 46,47), LSQISF (position 48–58), RGSNLTTH (position 59–66), PLRN (position 67–70), and IKDLVYMGNITIGTP (position 71–81) which are found to be conserved across ruminant PAG proteins. In general, the conserved regions are ones that are internal and structurally important for retaining the overall three-dimensional fold of the molecule. By contrast, the hypervariable regions are exposed and generally correspond with surface loops. Though the physiological significance of these changes is presently unknown, these hypervariable regions represent surface domains where amino acid substitutions could occur with little threat to the structural integrity of the molecules [4, 19]. The buffalo mature peptide sequence also contains variable regions as in other species having profound nonsynonymous substitutions. There are mainly four variable regions at positions 101–117, 162–173, 241–248, and 331–350. Since these proteins belong to glycoprotein family, they possess distinct sites for glycosylation. The potential sites for glycosylation in deduced buffalo PAG-1 protein sequence are at positions 78–81, 127–129, and 250–300 [4, 20, 21] (Figure 7). 

Phylogenetic Analysis of the buffalo PAG-1 sequences with other domestic species revealed buffalo PAG-1 is more related with bovine, caprine, and ovine species and less similar to equine, porcine, deer, and zebra due to various nonsynonymous substitutions in the entire sequence in the latter species. Porcine PAG-1 forms an entire different clade as it consists of variant amino acids residues when compared with ruminant species. Phylogram constructed between buffalo PAG-1 and different bovine PAGs sequences revealed buffalo PAG-1 was more related with bovine PAG-1 thereby belonging to the recently duplicated PAG gene group (PAG-1 group) and differing significantly from the ancient PAG group consisting of PAG-2 and 8 [13–15].

## 4. Conclusion

Buffalo Pregnancy-Associated Glycoprotein-1 cDNA encoding buffalo PAG-1 protein of 380 amino acids was characterized and found to be recently duplicated group members of the aspartic proteinase family being proteolytically inactive due to key mutations close to the active site.
